# Optimization design of hump phenomenon of low specific speed centrifugal pump based on CFD and orthogonal test

**DOI:** 10.1038/s41598-022-16430-w

**Published:** 2022-07-15

**Authors:** Wang Yu-qin, Ding Ze-wen

**Affiliations:** 1 School of Mechanical Engineering of Chaohu University, Anhui Chaohu, 238000 China; 2 Technological University of the Philippines, 1106 Manila, Philippines

**Keywords:** Engineering, Materials science, Mathematics and computing, Optics and photonics

## Abstract

Aiming to eliminate the hump phenomenon in a low specific speed centrifugal pump, its structural parameters were optimized using the computational fluid dynamics method. Based on the $k - \varepsilon$ turbulence model, a 3D steady analysis of the internal flow field was carried out. The $L_{9} \left( {3^{4} } \right)$ orthogonal table was established, and four structural parameters, including the impeller outlet diameter, impeller outlet width, number of blades, and blade outlet angle, were selected as influencing factors. Nine orthogonal test schemes were developed and the results were analyzed through the weight matrix analysis method, obtaining the weight of the selected factors on the test results. The optimal scheme was selected according to the weight and the weight matrix analysis results have shown that the impeller outlet width had the dominant influence on the head, shaft power, and efficiency. Further, the blade number was the main influencing factor for shaft power and efficiency. The centrifugal pump flow control test bench was built to carry out the numerical simulation and test all the prototype and optimization pump indexes. Through the external characteristic test, it can be seen that the $\beta_{2} Z^{0.773}$ of the optimized pump is 87.889, which is 24.89% lower than that of the prototype pump, which effectively optimizes the hump phenomenon of the centrifugal pump. The experimental results have shown that in underrated working conditions, the working performance of the optimized pump was improved significantly. The head size was reduced by 1.424%, and the efficiency was increased by 7.896%. By optimizing the structural pump parameters, its jet-wake hydraulic loss was reduced, and the head curve hump phenomenon was effectively eliminated. All the performance indexes of the optimized pump were higher than those of the prototype, verifying both the accuracy and reliability of the orthogonal test and weight matrix analysis method. Finally, obtained results provide a reference for the structural design of high-performance centrifugal pumps.

## Introduction

A low specific speed centrifugal pump is a type of centrifugal pump with a specific speed ranging between 20 and 80. It is characterized by low flow, high head, and low volume, and is widely used in production and life^1^. Using a low specific speed centrifugal pump, it is easy to produce an unstable hump phenomenon under the low flow rate condition. This, in turn, increases vibration and noise, thus shortening the pump service life and reducing its working reliability. Currently, the hump phenomenon mechanism of low specific speed centrifugal pumps is not clear, and the hump phenomenon of the head curve cannot be eliminated from the design. Therefore, besides studying the centrifugal pump working mechanism, it is also necessary to optimize the critical pump structural parameters to improve its working performance.

For a long time, reducing and eliminating the hump phenomenon of centrifugal pump head curve with low specific speed becomes an important segment of centrifugal pump research. Zhang Desheng et al.^2^ established ten design schemes and performed numerical simulation and performance prediction on low specific speed centrifugal pumps. The authors obtained distributions of static pressure, streamlines, velocity, and turbulent kinetic energy in the pump, and improved the internal flow characteristics. Zhang et al.^3^ used the SAS turbulence model to carry out the full-channel numerical simulation of the pump-turbine, determining the effect of the pump flow structure mechanism on the hump characteristics. Zhang Peifang et al.^4^ analyzed the hydraulic performance of low specific speed centrifugal pumps, observing the cause of the head curve hump phenomenon aiming to propose a solution. Li et al.^5^ used the three-dimensional constant value simulation equation to design the pump blades. Further, they analyzed the impeller flow characteristics and found the pump energy discharge characteristics in the hump zone mode. Chen et al.^6^ experimented by adding two partitions in the pump suction section. Experimental results have shown that the proposed method could effectively improve the IS centrifugal pump performance curve and eliminate the head curve hump.

With the continuous development of computational fluid dynamics (CFD), numerical simulations have become one of the most important methods to study the internal flow field of centrifugal pumps^7–14^. In this paper, a subtype of the centrifugal pump was used as the research object, and the CFD method was used to calculate the internal flow field of the centrifugal pump. Accounting for the influence of various structural parameters on the pump performance, structural parameters were optimized via orthogonal tests. The centrifugal pump head, shaft power, and efficiency were calculated using range analysis. The optimal parameter combination was obtained through weight matrix analysis. A centrifugal pump flow control test bench was built to verify the design method. Finally, the experimental results have confirmed that the centrifugal pump performance indicators improved after carrying out the surface optimization; the head curve humps were eliminated, and the desired effect was achieved.

### Hydraulic design and theoretical analysis

#### Hydraulic design

The structural parameters of a centrifugal pump are as follows: flow $Q = 102{\text{m}}^{3} /h$, head $H = 100{\text{m}}$, rotational speed $n = 2900{\text{r}} /min$, and specific speed $n_{s} = 56.343$. The semi-open impeller structure was adopted and the main structural parameters included: blade number $Z = 7$, blade outlet angle $\beta_{2} { = }26^\circ$, blade wrap angle $\varphi { = }110^\circ$, impeller inlet $D_{j} = 100{\text{mm}}$ , and outlet $D_{2} = 290{\text{mm}}$ diameters, impeller outlet width $b_{2} = 13{\text{mm}}$, and volute base circle diameter $D_{3} = 302{\text{mm}}$.

**Figure 1:**
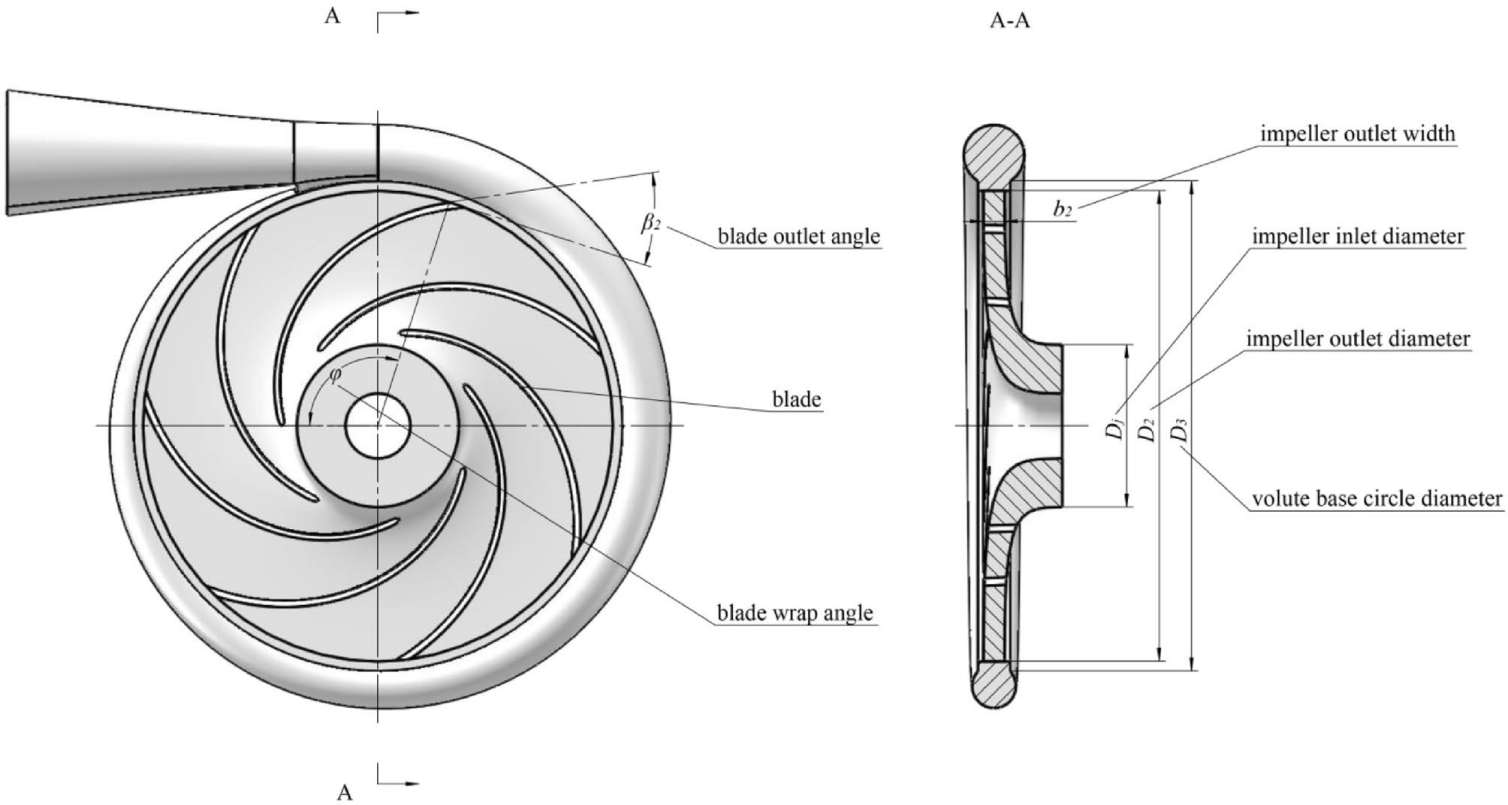
Calculation domain.

The centrifugal pump calculation domain is shown in Fig. 1. The fluid flows into the centrifugal pump along the $z$ axis, while the volute circumference is located in the $xoy$ plane. The overall calculation domain includes both the impeller and volute water body.

#### Theoretical analysis

During the operation, the centrifugal pump is affected by the impact loss. There is a difference between the theoretical $H_{t} - Q_{t}$ and the actual $H - Q$ centrifugal pump performance curves. According to theoretical analysis, theoretical centrifugal pump head $H_{t}$ and flow $Q_{t}$ satisfy the following equation:

1 $$ H_{t} = \frac{{u_{2} }}{g}\left( {\sigma u_{2} - \frac{{Q_{t} }}{{F_{2} }}\cot \beta_{2} } \right) $$

where $u_{2}$ is the circumferential blade outlet velocity calculated as:

2 $$ u_{2} = \frac{{D_{2} \pi n}}{60} $$

Further, in Eq. (1), $\sigma$ represents the Stoddard slip coefficient obtained as:

3 $$ \sigma = 1 - \frac{\pi }{Z}\sin \beta_{2} $$

By sorting equations given above, theoretical centrifugal pump head $H_{t}$ and flow $Q_{t}$ can be obtained via:

4 $$ H_{t} = \frac{{D_{2} \pi n}}{60g}\left[ {\left( {1 - \frac{\pi }{Z}\sin \beta_{2} } \right)\frac{{D_{2} \pi n}}{60} - \frac{{Q_{t} }}{{2\pi R_{2} b_{2} \psi_{2} }}\cot \beta_{2} } \right] $$

According to Eq. (4), the theoretical centrifugal pump head $H_{t}$ and flow $Q_{t}$ have a linear relationship. The theoretical $H_{t} - Q_{t}$ curve shows a gentle trend, while the actual $H - Q$ curve is prone to the hump phenomenon^15^. When aiming to find the curve without a hump phenomenon, its slope should be increased. Using Eq. (4), impeller outlet diameter, impeller outlet width, the number of blades, and blade outlet angle were selected as critical factors affecting the hump phenomenon in the actual $H - Q$ curve.

### Orthogonal test scheme design and numerical calculation

#### Orthogonal Test Factors

Under the small flow conditions, a low specific speed centrifugal pump easily generates vortex inside the flow passage which blocks the flow, causing large hydraulic loss and head reduction. This makes it easy to produce the hump phenomenon in the $H - Q$ curve.

An orthogonal test is an analysis method for studying the influence of multiple factors and levels on test results by using an orthogonal table^16^. Using the orthogonality, representative combinations can be selected to experiment, and the influence of each parameter is analyzed to obtain the optimal combination. Thus, the orthogonal test is an efficient, fast, and economical method for designing experiments.

Based on the $H_{t} - Q_{t}$ curve equation analysis and by considering the influence of various structural parameters, the impeller outlet diameter, its width, blade number, and blade outlet angle were selected as the experimental factors for the orthogonal test. The orthogonal test table was used to combine all the factors and carry out the numerical calculation, among which three horizontal values were set for each factor, as shown in Table 1.

**Table 1:** Factor level table.

Levels	Factors			
	$A$	$B$	$C$	$D$
	$D_{2} /{\text{mm}}$	$b_{2} /{\text{mm}}$	$Z$	$\beta_{2} /^\circ$
1	290	13	5	24
2	295	11	6	26
3	300	9	7	22

According to $L_{9} (3^{4} )$ the orthogonal test table, nine test scheme groups were set up (see Table 2).

**Table 2:** Orthogonal test table.

Serial numbers	Combinations	Corresponding parameters			
		$D_{2} /{\text{mm}}$	$b_{2} /{\text{mm}}$	$Z$	$\beta_{2} /^\circ$
1	$A_{1} B_{1} C_{1} D_{1}$	290	13	5	24
2	$A_{1} B_{2} C_{2} D_{2}$	290	11	6	26
3	$A_{1} B_{3} C_{3} D_{3}$	290	9	7	22
4	$A_{2} B_{1} C_{2} D_{3}$	295	13	6	22
5	$A_{2} B_{2} C_{3} D_{1}$	295	11	7	24
6	$A_{2} B_{3} C_{1} D_{2}$	295	9	5	26
7	$A_{3} B_{1} C_{3} D_{2}$	300	13	7	26
8	$A_{3} B_{2} C_{1} D_{3}$	300	11	5	22
9	$A_{3} B_{3} C_{2} D_{1}$	300	9	6	24

#### Grid division

The GAMBIT professional grid generation software was used to divide the computational domain and obtain high-quality hexahedral grids. Additionally, it was used to refine local grids for complex structures. Four groups of grids with rather various grid numbers were selected based on head to carry out the grid independence analysis and to ensure the reliability and accuracy of the calculation results. The results are shown in Table 3. When the number of grids exceeded 2.14 million, the calculated head changed slightly, proving that grids are not relevant. Thus, the final number of grids was 2.14 million.

**Table 3:** Grid independence analysis.

Programmes	Grid numbers $/10^{4}$	$H/m$
Grid 1	172	116.7332
Grid 2	214	115.1091
Grid 3	268	115.0912
Grid 4	312	115.1068

**Figure 2:**
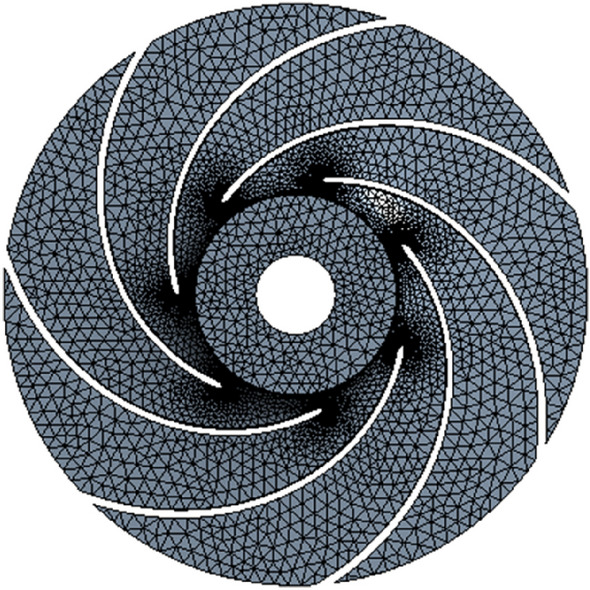
Impeller grids.

**Figure 3:**
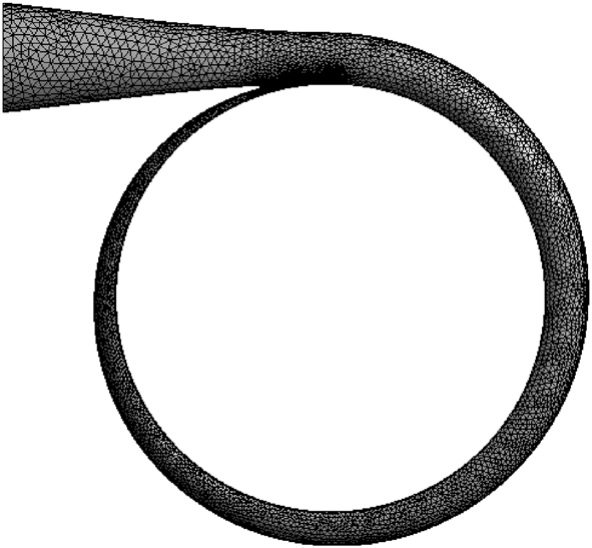
Volute grids.

Figures 2 and 3 show schematic diagrams of calculation domain grids.

#### Boundary conditions

The internal flow field was calculated using Fluent software. The numerical solution was calculated based on the finite volume method of the completely unstructured grid. The standard $k - \varepsilon$ model was selected as the turbulence model to solve the internal flow field. Moreover, the SIMPLE algorithm was used as the coupling method for internal pressure and velocity. The speed inlet was taken as the inlet condition, while the outlet boundary condition was free outflow. The non-slip wall was taken as the fixed-wall boundary condition, and its rotating counterpart was taken as the impeller wall. The convergence accuracy was 0.0001.

### Weight matrix optimization design

#### Orthogonal test results

The CFD software was used to simulate the internal flow field of nine test combination groups. A mathematical model using head, shaft power, and efficiency as objective functions was established. Their respective values were calculated respectively using Eq. (5)^17^. The calculation results are shown in Table 4.

5 $$ \left\{ {\begin{array}{*{20}c} {H = \frac{\Delta P}{{\rho g}}} \\ {P = M\omega } \\ {\eta = \frac{QHg}{{3.6 \times 10^{6} \times P}}} \\ \end{array} } \right.. $$

**Table 4:** Orthogonal test results.

Serial numbers	$H/m$	$P/{\text{kw}}$	$\eta /{\text{\% }}$
1	102.3664878	35.68880796	79.6433477
2	104.4168653	35.36858793	81.9741036
3	100.5910882	33.97658865	82.2059932
4	114.795951	39.69268193	80.3044982
5	112.1100155	38.40383611	81.10575647
6	97.40709746	32.85840294	82.3138348
7	124.8159102	43.902878	78.940651
8	107.7217253	36.34234605	82.3027009
9	108.2402631	35.70413202	84.1771284

#### Range analysis

Range analysis was carried out using the orthogonal test results; the analysis results are shown in Table 5.

**Table 5:** Range analysis results.

Parameters		$D_{2} /{\text{mm}}$	$b_{2} /{\text{mm}}$	$Z$	$\beta_{2} /^\circ$
$H/m$	$k_{1}$	102.4581471	113.992783	102.4984369	107.5722555
	$k_{2}$	108.1043547	108.0828687	109.1510265	108.8799577
	$k_{3}$	113.5926329	102.0794829	112.5056713	107.7029215
	$s$	11.13448577	11.91330008	10.00723445	1.307702183
$P/{\text{kw}}$	$k_{1}$	35.01132818	39.76145596	34.96318565	36.59892536
	$k_{2}$	36.98497366	36.70492336	36.92180063	37.37662296
	$k_{3}$	38.64978536	34.17970787	38.76110092	36.67053888
	$s$	3.638457177	5.581748093	3.79791527	0.777697593
$\eta /{\text{\% }}$	$k_{1}$	81.2744815	79.62949897	81.41996113	81.64207752
	$k_{2}$	81.24136316	81.79418699	82.15191007	81.07619647
	$k_{3}$	81.80682677	82.89898547	80.75080022	81.60439743

As shown in Table 5, when considering head, the main sequence of influencing indexes was $BACD$, with the optimal combination as follows: $D_{2} { = }300{\text{mm}}$, $b_{2} { = }13{\text{mm}}$, $z{ = }7$, and $\beta_{2} = 26^\circ$. For shaft power, the main sequence of influencing indexes was $BCAD$ and the optimal combination was $D_{2} { = }290{\text{mm}}$, $b_{2} { = }9{\text{mm}}$, $z{ = }5$, and $\beta_{2} = 24^\circ$. Finally, for the efficiency, influencing indexes of various factors were arranged as $BCDA$, with combination $D_{2} { = }300{\text{mm}}$, $b_{2} { = }9{\text{mm}}$, $z{ = }6$, and $\beta_{2} = 24^\circ$ being optimal. Aiming to rapidly find the optimal combination and the main sequence of influencing indexes for each factor, weight matrix analysis was carried out. The weight matrix was obtained for three objective functions and structural parameters were selected according to the weight.

#### Weight matrix analysis

The multi-objective optimization of the weight matrix was carried out to establish a three-layer structure model based on the orthogonal test scheme^18^, as shown in Table 6.

**Table 6:** Data structure of weight matrix analysis.

First layer	Objective function							
Second layer	$A_{1}$			…	…	$A_{l}$		
Third layer	$A_{11}$	…	$A_{1m}$	…	…	$A_{l1}$	…	$A_{lm}$

The first layer represents the test objective function layer and is defined; assuming there are $l$ influencing factors in the orthogonal test, each influencing factor has $m$ levels. Moreover, the objective function value of the factor $A_{i}$ at level $j$ is $k_{ij}$. The larger value of the orthogonal experiment objective function represents a better specimen; hence $K_{ij} = k_{ij}$. If the objective function value is smaller, the better, then $K_{ij} = 1/k_{ij}$. The matrix was established, as shown in Eq. (6).

6 $$ M = \left[ {\begin{array}{*{20}c} {K_{11} } & 0 & \cdots & 0 \\ {K_{12} } & 0 & \cdots & 0 \\ \cdots & \cdots & \cdots & \cdots \\ {K_{1m} } & 0 & \cdots & 0 \\ 0 & {K_{21} } & \cdots & 0 \\ 0 & {K_{22} } & \cdots & 0 \\ \cdots & \cdots & \cdots & \cdots \\ 0 & {K_{2m} } & \cdots & 0 \\ \begin{gathered} \cdots \hfill \\ 0 \hfill \\ \end{gathered} & \begin{gathered} \cdots \hfill \\ 0 \hfill \\ \end{gathered} & \begin{gathered} \cdots \hfill \\ \cdots \hfill \\ \end{gathered} & \begin{gathered} \cdots \hfill \\ K_{l1} \hfill \\ \end{gathered} \\ \cdots & \cdots & \cdots & \cdots \\ 0 & 0 & \cdots & {K_{l2} } \\ 0 & 0 & \cdots & {K_{lm} } \\ \end{array} } \right] $$

The second layer represents all the factor layers and is defined as $T_{i} = 1/\sum\nolimits_{j = 1}^{m} {K_{ij} }$. The matrix shown in Eq. (7) was established next:

7 $$ T = \left[ {\begin{array}{*{20}c} {T_{1} } & 0 & \cdots & 0 \\ 0 & {T_{2} } & \cdots & 0 \\ \cdots & \cdots & \cdots & \cdots \\ 0 & 0 & \cdots & {T_{l} } \\ \end{array} } \right] $$

The third layer is horizontal; the extreme differences in values of the orthogonal test factors were denoted as $s_{i}$, which is defined as $S_{ij} = s_{i} /\sum\nolimits_{i = 1}^{l} {s_{i} }$. Finally, the matrix shown in Eq. (8) can be written:

8 $$ S = \left[ {\begin{array}{*{20}c} {S_{1} } \\ {S_{2} } \\ \cdots \\ {S_{l} } \\ \end{array} } \right] $$

The weight matrix affects the objective function and is defined as: $\omega = MTS$ and was established as:

9 $$ \omega^{T} = \left[ {\omega_{1} ,\omega_{2} , \ldots ,\omega_{m} } \right] $$

In Eq. (9), $\omega_{1} = K_{11} T_{1} S_{1}$, where $K_{11} T_{1}$ is the ratio between the factor $A_{1}$ target value at the first level to the target value of all the levels. Further, $S_{1}$ is the ratio between the polar difference of factor $A_{1}$ and the total polar difference. The product result reflects the influence of factors $A_{1}$ on the objective function at the first level, along with the magnitude of extreme factor $A_{1}$ differences. Through weight matrix analysis, the influence of each factor level on the objective function can be obtained. The primary and secondary order and the optimal combination of factor influences on the objective function can be quickly obtained through weight.

Using the above-presented expressions, weight matrices of three objective functions were calculated. For the head and efficiency, the higher objective function values represent the better solution, meaning that the corresponding values are $K_{ij} = k_{ij}$, $T_{i} = 1/\sum\nolimits_{j = 1}^{m} {K_{ij} }$, $S_{ij} = s_{i} /\sum\nolimits_{i = 1}^{l} {s_{i} }$. For the shaft power, a lower objective function value is better. The corresponding values are $K_{ij} = 1/k_{ij}$, $T_{i} = 1/\sum\nolimits_{j = 1}^{m} {K_{ij} }$, $S_{ij} = s_{i} /\sum\nolimits_{i = 1}^{l} {s_{i} }$.

The first objective function weight matrix (head) is as follows:

$$ \omega_{{1}} { = [}0.1024,0.1081,0.1135,0.1219,0.1156,0.1092,0.0921,0.0981,0.1011,0.0126,0.0128,0.0126{]}^{{\text{T}}} $$

The second objective function weight matrix (shaft power) is as follows:

$$ \omega_{2} { = [}0.0925,0.0875,0.0838,0.1246,0.1350,0.1450,0.0966;0.0915,0.0872,0.0189,0.0185,0.0189{]}^{{\text{T}}} $$

The third objective function weight matrix (efficiency) is:

$$ \omega_{3} { = [}0.0324,0.0324,0.0326,0.1837,0.1887,0.1912,0.0805,0.0812,0.0798,0.0326,0.0324,0.0326{]}^{{\text{T}}} $$

Finally, the total weight matrix of the orthogonal test objective function is the average of the weight matrices of three objective functions:

$$ \omega_{avg} { = [}0.0758,0.0760,0.0766,0.1434,0.1464,0.1485,0.0897,0.0903,0.0894,0.0214,0.0212,0.0214{]}^{{\text{T}}} $$

According to the weight matrix calculation results, factor influences on the orthogonal test objective function are ordered as $BCAD$; weights of the horizontal values for each factor are $A_{3}$, $B_{3}$, $C_{2}$, and $D_{3}$. The optimal orthogonal test combination scheme is $A_{3} B_{3} C_{2} D_{3}$, namely: $D_{2} { = }300\,{\text{mm}}$, $b_{2} { = }9\,{\text{mm}}$, $Z{ = }6$, and $\beta_{2} = 22^\circ$.

#### Internal flow field analysis

To verify the optimization model feasibility, the full-flow CFD numerical simulation of the optimization model was carried out. The outputs were compared to results for the prototype pump.

**Figure 4:**
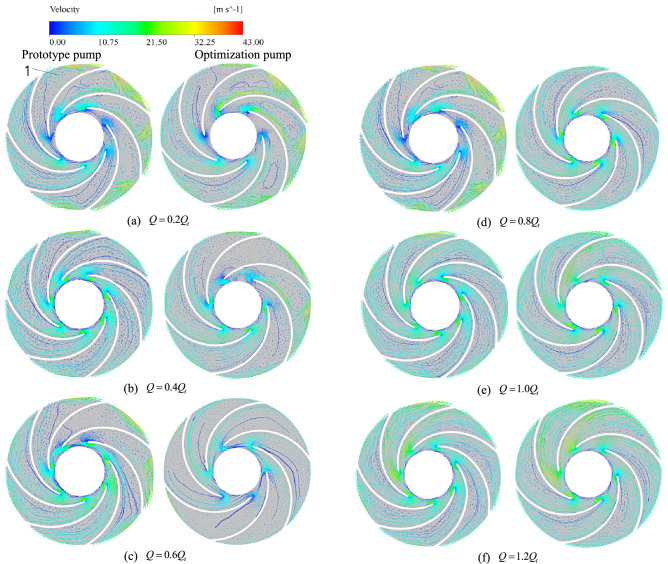
The relative velocity distribution at the intermediate interface of centrifugal pump.

Counter-clockwise numbering was used near the tongue flow passage (No. 1 flow passage). The internal pressure surface speed of each flow passage was greater than the suction surface speed. Additionally, the outlet speed reached the maximum value in the last flow passage. Due to the hydraulic impact between the flow passage and the tongue, the liquid flow direction changed. The relative velocity distribution at the intermediate centrifugal pump interface is shown in Fig. 4.

When the small flow rate condition is active, the low-speed zone is mainly found on the blade pressure surface. Simultaneously, the liquid flow direction is disorderly between the blade inlet and outlet. Once the flow rate drops under $0.8Q_{t}$, the degree of turbulence increases continuously. The vortices appear in multiple flow channels, and the number of vortices increases with the decrease in flow rate. Vortices within flow passages are continuously expanding in the clockwise impeller direction, blocking by low-speed liquid. This results in larger hydraulic losses, causing the prototype pump to produce a hump of centrifugal pump head curve under the condition of a small flow rate.

For the optimization pump, the degree of liquid flow direction disorder can be optimized above the $0.4Q_{t}$ flow condition. In that case, the liquid flow direction in each impeller passage is more stable. Furthermore, under the $0.4Q_{t}$ flow condition, a small number of vortices appear in the flow passage, similar to the prototype pump under the small flow condition. Thus, the head located at the closed dead center of the optimization pump is decreased.

### Experimental verification

**Figure 5:**
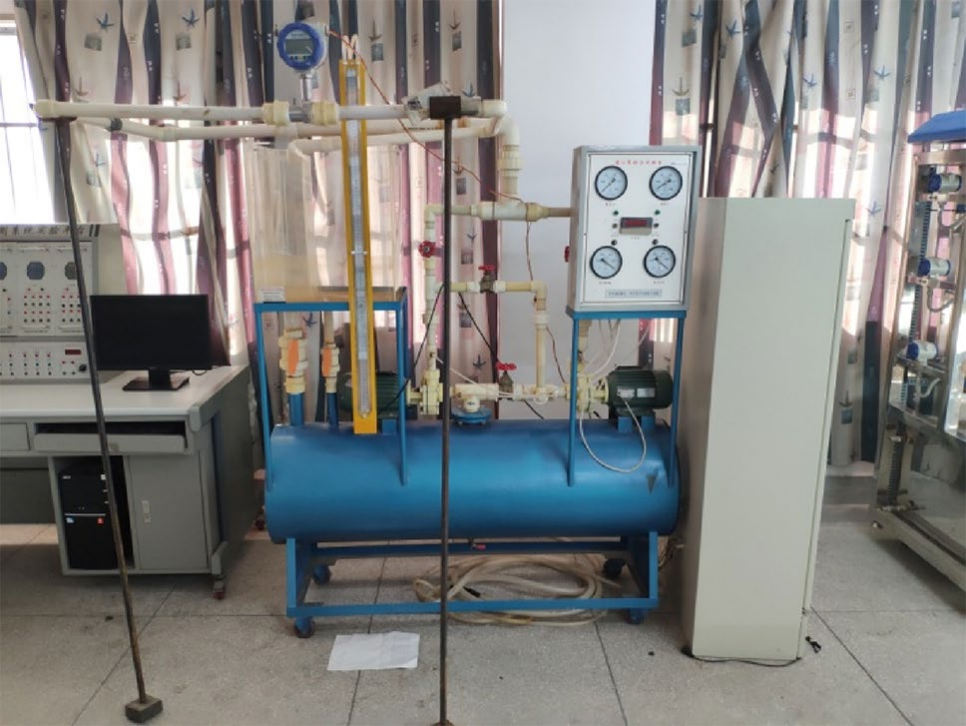
Flow control test bench.

A flow control test bench was built based on the NGL002 centrifugal pump test device to verify the accuracy of optimization results, as shown in Fig. 5. The prototype pump and the optimization pump were tested and verified for multi-condition external characteristics.

**Figure 6:**
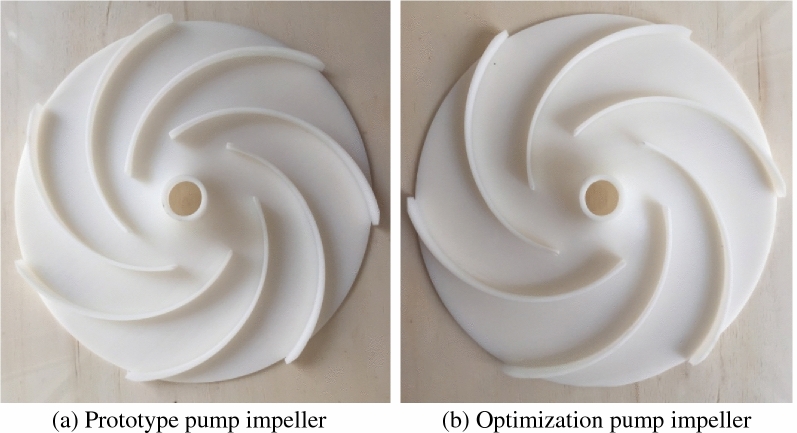
Impeller.

The prototype and the optimization pump impellers are shown in Fig. 6.

**Figure 7:**
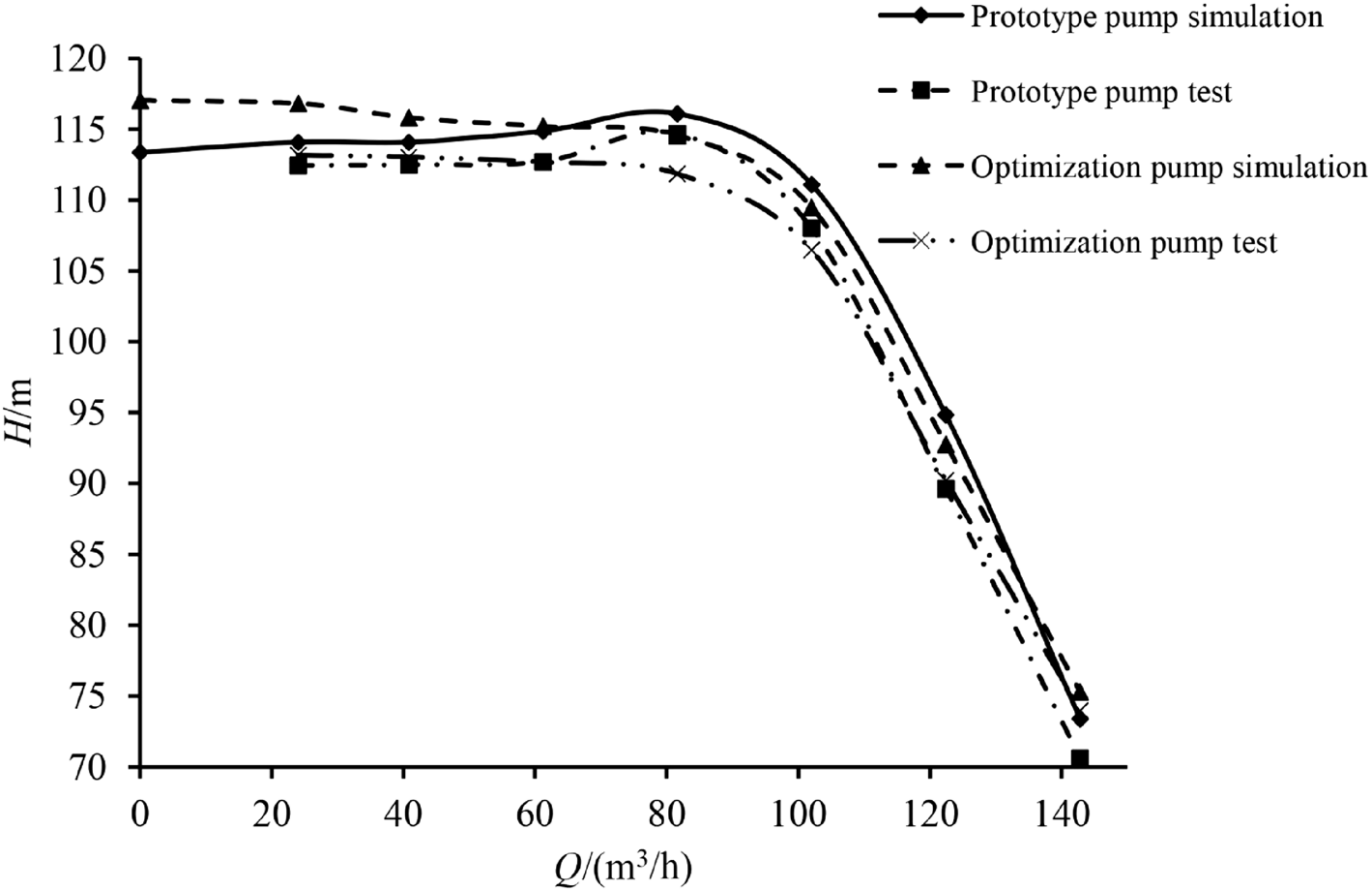
Head curve.

**Figure 8:**
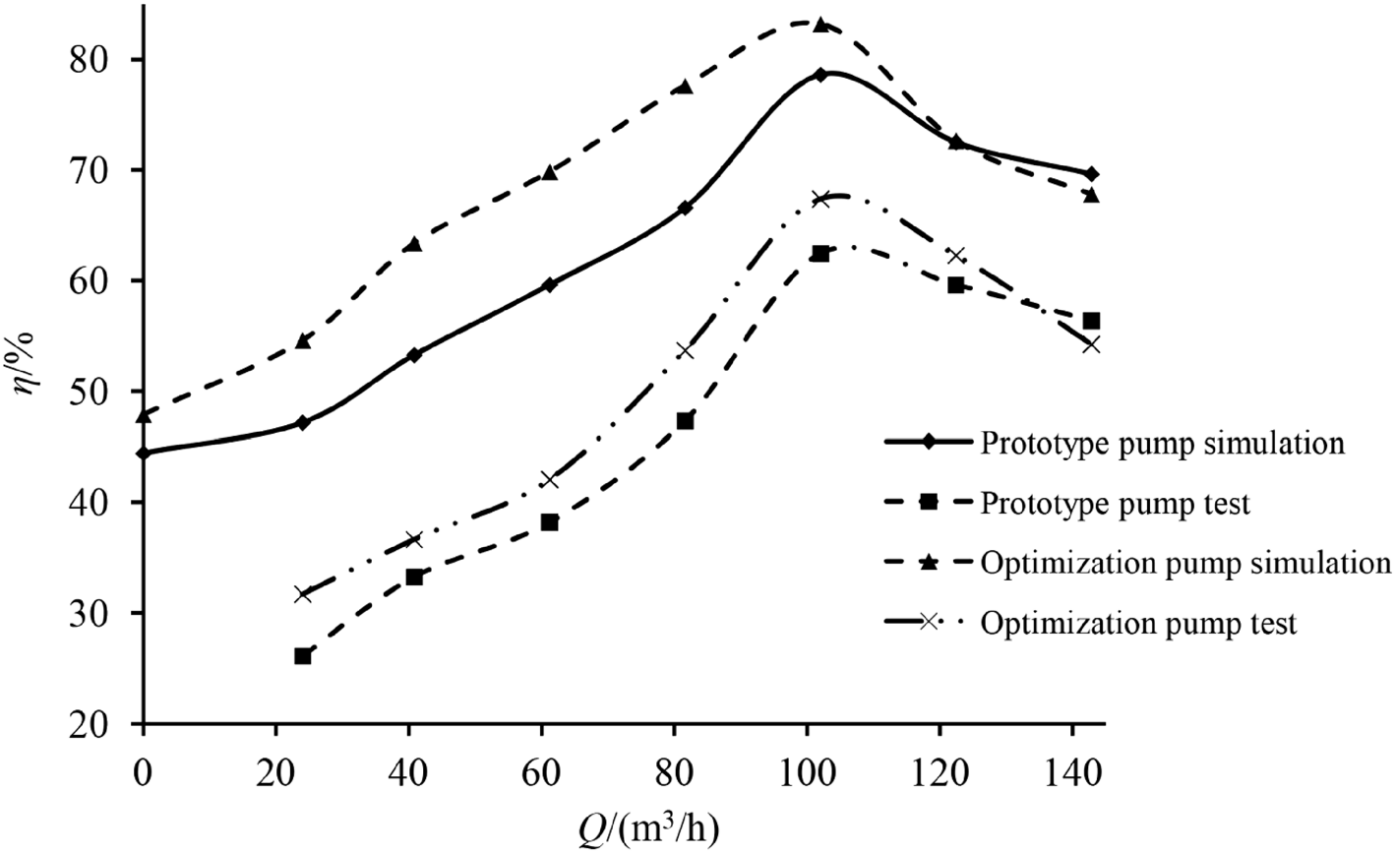
Efficiency curve.

The experimental results containing the prototype and the optimization pump external characteristics are shown in Figs. 7 and 8.

As can be seen from the test results, the variation law of the external characteristic curve practically remains the same despite varying working conditions. The numerical simulation results are in good agreement with the experimental values, reflecting the changing trend of all indexes under different flow rates. The head and efficiency obtained via numerical simulation are higher than the experimental values, mostly because the energy loss of each part and the impeller manufacturing errors were not considered in the numerical simulation. Using the verification results obtained for external characteristics, it can be seen that the optimization pump performance index is higher than that of the prototype. The $\beta_{2} Z^{0.773}$ prototype pump and optimization pump are 117.013 and 87.889, respectively. Since the optimized blade outlet angle and number of blades are reduced, the hump phenomenon was effectively optimized^19^.

The external characteristic test results were extracted for various indexes, as shown in Table 7.

**Table 7:** Test results of each index.

Indexes	$Q/({\text{m}}^{3} /h)$						
	24	40.8	61.2	81.6	102	122.4	142.8
Prototype pump $H/{\text{m}}$	112.458	112.506	112.711	114.694	108.021	89.627	70.637
Optimization pump $H/{\text{m}}$	113.187	113.045	112.687	111.869	106.483	90.216	73.996
$\Delta H/(\% )$	0.648	0.479	− 0.021	− 2.463	− 1.424	0.657	4.755
Prototype pump $\eta /\%$	26.13	33.28	38.21	47.33	62.44	59.62	56.37
Optimization pump $\eta /\%$	31.71	36.65	42.06	53.68	67.37	62.28	54.24
$\Delta \eta /(\% )$	21.355	10.126	10.076	13.416	7.896	4.462	− 3.779

Based on Table 7, it is evident that under rated conditions, the head of the optimized centrifugal pump was reduced by 1.424%, while the efficiency increased by 7.896%. As the prototype pump produced a hump under $0.8Q_{t}$ working conditions, the head under $0.6Q_{t} - 1.0Q_{t}$ working conditions was greater than the optimization pump head. The actual optimization pump $H - Q$ curve does not show a hump phenomenon, meaning that the optimization goal is achieved: the index values are effectively improved. To sum up, the orthogonal test and weight matrix analysis method are feasible, with the optimized design scheme accuracy being verified.

## Conclusion

1. Hydraulic design of low specific speed centrifugal pump was carried out. Three-dimensional impeller and volute models were created.
2. Nine groups of test schemes were designed using the orthogonal test, and the influence order of each factor on each of the indexes was obtained through range analysis. Further, by using the weight matrix analysis and the weight relationship between the factor levels, a set of optimization models were obtained: $D_{2} { = }300{\text{mm}}$, $b_{2} { = }9{\text{mm}}$, $Z{ = }6$, and $\beta_{2} = 22^\circ$. The internal flow field of the prototype and the optimization pump was numerically simulated. The simulation results have shown that the hump phenomenon was significantly improved in the optimization pump. Hence, the feasibility of the optimization scheme was verified.
3. The centrifugal pump flow control test bench was built and the simulation and test values of each prototype and optimization pump index were obtained under different working conditions. Test results have shown that the performance indexes were higher in the optimization pump, eliminating the hump phenomenon and improving its hydraulic performance. Accuracies of the design process and optimization method were further verified.
4. In this paper, a type of centrifugal pump was taken as the research object and hydraulic design was carried out based on the CFD. The main structural parameters were optimized via an orthogonal test. A test platform was built to enable carrying out verification tests, which verified the orthogonal test scheme accuracy and improving the working performance of the centrifugal pump. Compared to the prototype pump, the outlet impeller diameter of the optimized pump was increased, while the impeller outlet width, the number of blades, and the blade outlet angle were smaller. With the increase in the impeller outlet diameter and the decrease of its width, the flow passage area increased, reducing the hydraulic losses. In that case, the overload would be avoided, and the "jet-wake" phenomenon and hump would be eliminated. At the same time, with the decrease in the number of blades and the blade outlet angle, the area of each flow channel increased. Thus, the static pressure at the blade outlet decreased, mostly to avoid local backflow and flow separation. It can be seen that, by optimizing the structural centrifugal pump parameters, the pump head was reduced, and the internal flow stability and working efficiency were improved. Thus, a series of negative problems such as vibration, noise, and high energy consumption of pump and pipeline systems caused by the hump phenomenon could be mitigated effectively. In the next step, multi-objective optimization design will be carried out around the external characteristics of centrifugal pump head, shaft power and efficiency, focusing on in-depth research on centrifugal pump flow-induced vibration noise, in order to comprehensively improve the working performance of centrifugal pump.
